# Correlation between the functional impairment of bone marrow-derived circulating progenitor cells and the extend of coronary artery disease

**DOI:** 10.1186/1479-5876-10-143

**Published:** 2012-07-09

**Authors:** Ilkay Bozdag-Turan, R Goekmen Turan, Lylia Paranskaya, Nicole S Arsoy, C Hakan Turan, Ibrahim Akin, Stephan Kische, Jasmin Ortak, H Schneider, S Ludovicy, Tina Hermann, Giuseppe D’Ancona, Serkan Durdu, A Ruchan Akar, Hueseyin Ince, Christoph A Nienaber

**Affiliations:** 1 Department of Internal Medicine, Division of Cardiology, University hospital Rostock, Ernst Heydemann Str 6, Rostock, 18055, Germany; 2 Stem Cell Institute, Ankara University, Ankara, Turkey

**Keywords:** Circulating progenitor cells, Migration capacity, Colony forming capacity, Ischemic heart disease, Diabetes, Coronary artery disease

## Abstract

**Background:**

Bone marrow-derived circulating progenitor cells (BM-CPCs) in patients with coronary heart disease are impaired with respect to number and functional activity. However, the relation between the functional activity of BM-CPCs and the number of diseased coronary arteries is yet not known. We analyzed the influence of the number of diseased coronary arteries on the functional activity of BM-CPCs in peripheral blood (PB) in patients with ischemic heart disease (IHD).

**Methods:**

The functional activity of BM-CPCs was measured by migration assay and colony forming unit in 120 patients with coronary 1 vessel (IHD1, n = 40), coronary 2 vessel (IHD2, n = 40), coronary 3 vessel disease (IHD3, n = 40) and in a control group of healthy subjects (n = 40). There was no significant difference of the total number of cardiovascular risk factors between IHD groups, beside diabetes mellitus (DM), which was significantly higher in IHD3 group compared to IHD2 and IHD1.

**Results:**

The colony-forming capacity (CFU-E: p < 0.001, CFU-GM: p < 0.001) and migratory response to stromal cell-derived factor 1 (SDF-1: p < 0.001) as well as vascular endothelial growth factor (VEGF: p < 0001) of BM-CPCs were reduced in the group of patients with IHD compared to control group. The functional activity of BM-CPCs was significantly impaired in patients with IHD3 as compared to IHD1 (VEGF: p < 0.01, SDF-1: p < 0.001; CFU-E: p < 0.001, CFU-GM: p < 0.001) and to IHD2 (VEGF: p = 0.003, SDF-1: p = 0.003; CFU-E: p = 0.001, CFU-GM: p = 0.001). No significant differences were observed in functional activity of BM-CPCs between patients with IHD2 and IHD1 (VEGF: p = 0.8, SDF-1: p = 0.9; CFU-E: p = 0.1, CFU-GM: p = 0.1). Interestingly, the levels of haemoglobin AIc (HbAIc) correlated inversely with the functional activity of BM-CPCs (VEGF: p < 0.001, r = −0.8 SDF-1: p < 0.001, r = −0.8; CFU-E: p = 0.001, r = −0.7, CFU-GM: p = 0.001, r = −0.6) in IHD patients with DM.

**Conclusions:**

The functional activity of BM-CPCs in PB is impaired in patients with IHD. This impairment increases with the number of diseased coronary arteries. Moreover, the regenerative capacity of BM-CPCs in ischemic tissue further declines in IHD patients with DM. Furthermore, monitoring the level of BM-CPCs in PB may provide new insights in patients with IHD.

## Background

Circulating progenitor cells (CPCs) are primitive bone marrow cells (BMCs), that have the capacity to proliferate, migrate, and differentiate into various mature cell types. [1-3] Furthermore, these cells circulate in peripheral blood, and implicate in neoangiogenesis after tissue ischemia. [4-6] Experimental studies have shown that re-introduction of cytokines such as vascular endothelial growth factor (VEGF), angiopoetin-1, SDF-1, G-CSF, or GM-CSF enhance the mobilization of the BM-CPCs to the ischemic myocardium, augmenting neovascularization. [7,8] It has been suggested that cardiovascular risk factors (CVRFs) are associated with reduction of functional activity of BM-CPCs in patients with coronary artery disease as well as in healthy men. [9,10] Moreover, especially diabetes has been shown to reduce numbers and impair functional activity of BM-CPCs. [11-13] However, it is unknown whether the functional activity of BM-CPCs relates to the number of diseased coronary arteries in patients with IHD. In this study, we analyzed the functional activity of BM-CPCs and their relationship with the number of diseased coronary arteries in IHD-patients.

## Methods

### Study protocol and study population

132 IHD patients between 18–80 years of age were screened for inclusion in this study, if they had had a documented MI at least 6 months ago and had left ventricular dysfunction. 12 of 132 patients had to be excluded from the study due to acute coronary syndrom and/or acutely decompensated heart failure. All 120 IHD patients underwent diagnostic cardiac catheterization due to stable angina. We selected a control group of 40 healthy subjects without overt heart disease and/or major cardiovascular risk factors such as diabetes, smoking, hypertension, hypercholesterolemia, and positive family history concerning IHD. All of them had atypical chestpain but no evidence of cardiac ischaemia. Control subjects underwent coronary angiography to rule out ischaemic heart disease within 24 hours after admission. The patients were recruited during diagnostic cardiac catheterization by interventional cardiologist and were separated into 4 groups (I) IHD1; II) IHD2; III) IHD3 and IV) Control group). After this step a peripheral blood sample was taken during cardiac catheterization to measure functional activity and characterization of BM-CPCs before any interventional procedure. A CVRFs score including age > 40 years, male sex, hypertension, diabetes, smoking, positive family history and hypercholesterolaemia was calculated according to Vita et al. [14] Hypertension was defined as a history of hypertension for >1 year that required the initiation of antihypertensive therapy by the primary physician. Smoking was defined as patients revealing a history of smoking for >2 pack-years and current smoking. Positive family history was defined as documented evidence of coronary artery disease (CAD) in a parent or sibling before 60 years of age. Hypercholesterolaemia was defined as fasting low-density-lipoprotein (LDL) cholesterol levels exceeding 130 mg/dl. Diabetes was defined as the need for oral antidiabetic drug therapy or insulin use.

Exclusion criteria were the presence of acutely decompensated heart failure with a New York Heart Association (NYHA) class of IV, infectious or inflammatory disease, surgery or trauma within 2 months, renal or liver dysfunction, thrombocytopenia, anemia, severe comorbidity and alcohol or drug dependency, history of other severe chronic diseases or cancer, or unwillingness to participate. The study conforms with the principles outlined in the Declaration of Helsinki and was approved by the local ethics committee. Written informed consent was obtained from each patient.

### Coronary angiography and left ventriculography

All IHD patients underwent left heart catheterization, left ventriculography and coronary angiography. Cardiac catheterization was performed according to the guidelines for coronary angiography of the American College of Cardiology and the American Heart Association. [15] Cardiac function was determined by left ventriculography. Cardiac function was evaluated by global EF. Global EF was measured with Quantcor software (Siemens, Erlangen/Germany). The extent of coronary artery disease was scored, by at least two independent interventional cardiologists, as 0 (stenosis < 50 percent), 1 (stenosis of any main coronary artery > 50 percent), 2 (stenosis of two main coronary arteries > 50 percent), and 3 (stenosis of three main coronary arteries > 50 percent).

### Phenotypic characterization of BM-CPCs

Phenotypic characterization of BM-CPCs were performed in 5 of 20 ml peripheral blood (PB) for CD34/45^+^ and CD133/45^+^ by flow cytometry (EPICS-XL, Beckmann Coulter).

Samples were stained with fluorescein isothiacyanate (FITC) conjugate of a CD45^+^ antibody (clone J33, Coulter/Immunotech, Marseille/France) that detects all isoforms and glycoforms of the CD45 family, phycoerythrin (PE) conjugate of a CD34^+^ antibody (clone 581, Coulter/Immunotech, Marseille/France) that detects a class III epitope on all glycoforms of the CD34^+^ antigen and PE conjugate of a CD133/1^+^ (Miltenyi Biotec, Bergisch Gladbach/Germany). Control samples were stained with CD45^+^ FITC and an IgG1 PE (Coulter/Immunotech, Marseille/France) isotype.

For each patient EDTA blood samples were labelled with CD34/45^+^, CD133/45^+^, and IgG1/CD45. All tubes were incubated at room temperature in the dark. After incubation, cells were lysed with ammonium chloride, washed with phosphate-buffered saline (PBS). Samples were then stored on ice at 4 °C in dark environment for 20 minutes and analysed by flow cytometry.

Samples were subjected to a 2D side scatter-fluorescence dot plot analysis. After appropriate gating, the concentration of BM-CPCs with low cytoplasmic granularity (low side ward scatter) was quantified and expressed as concentration of cells per million white blood cells.

### Isolation and cultivation of BM-CPCs

20 ml peripheral venous blood was taken using a BD Vacutainer CPT^TM^ from each patient. BM-CPCs were isolated by density gradient centrifugation. After 2 washing steps, cells were resuspended in 1 ml EBM2-medium (Cell system). The number of isolated BM-CPCs were determined in a Neubauer chamber.[10,16]

### Assessment of migration assay

A total of 1x10^6^ BM-CPCs were resuspended in 250 μl X-Vivo and placed in the top compartment of a Boyden Chamber. This chamber was placed in a 24-well culture dish contained either only EBM-2 medium or 100 ng/ml stromal cell derived factor-1 (SDF-1) or 100 ng/ml vascular endothelial growth factor (VEGF) in EBM2-medium. After 24 hours of incubation at 37 °C transmigrated cells were counted by 2 independent investigators. [10,16] Quantitative evaluation of migrated cells to SDF-1 and VEGF were analyzed in comparison to cells without chemokine in blood samples of both groups. Values are expressed as % of migrated cells without chemokine.

### Assessment of colony forming unit assay

1x10^5^ in BM-CPCs per ml were seeded in Methocult GF H4435 (Stemcell Technologies). Culture dishes were seeded with 1 ml cell suspension and then incubated at 37 °C. Colony-forming unit erythroid (CFU-E) and CFU-granulocyte/macrophage (CFU-GM) were studied under phase-contrast microscopy and were counted after 14 days of incubation by 2 independent investigators. [16]

### Biochemical measurements

Peripheral blood was collected from patients with IHD and control group on day 1 of admission. Serum creatine phosphokinase (CPK) values (normal range: 24–195 U/l), inflammatory markers such as C - reactive protein (normal range <0.5 mg/dl) and leukocytes (normal range: 4–12 x10^3^/μl) and routine laboratory tests with HbA1C were measured in the study population.

### Statistical analysis

Continuous data are presented as mean ± SD. Comparison of the distributions of a continuous variable between two independent groups was performed using the two-sided nonparametric Mann–Whitney test. The type I error rate α was chosen as 5% and two-sided p-values equal or less 0.05 were interpreted as statistically significant. Some qualitative baseline characteristics were compared using the Fisher's Exact-Test.

Bivariate regression analysis was presented in a graphical form and Pearson's correlation coefficient was obtained.

Statistical significant was accepted, if the corresponding two-sided p-value was smaller or equal to 0.05. Statistical analysis was performed with SPSS for Windows (Version 15.0).

## Results

### Baseline characteristics of the patients and healthy control subjects

We included 120 patients with IHD and 40 healthy subjects without heart disease in the study. The baseline characteristics of the study population are depicted in Table 1. Although there was no significant difference in total number of CVRFs between all IHD patients groups, interestingly, there was a significant difference in the number of DM patients in IHD3 group compared to IHD1 and IHD2. In contrast, there was no significant difference in the number of DM patients between IHD1 and IHD2. Also no significant differences were observed in other baseline characteristics and demographics of patients between all IHD groups. No significant differences were observed in age and sex between all groups (Table 1).

**Table 1:** Baseline clinical characterics of the study population

			**IHD1(n = 40)**	**IHD 2(n = 40)**	**IHD 3(n = 40)**	**P**	**Control Group (n=40)**
Age			60 ± 15	60 ± 11	64 ± 10	NS	66 ± 10
male			24	26	25	NS	21
Cardiovascular Risk Factors % (n)							
	Hypertension		80 (n = 32)	80 (n = 32)	78 (n = 31)	NS	-
	Hyperlipidemia		60 (n = 24)	60 (n = 24)	53 (n = 21)	NS	-
	Smoking		68 (n = 27)	75 (n = 30)	63 (n = 25)	NS	-
	Positive family history of CAD		40 (16)	40 (n = 16)	35 (n = 14)	NS	-
	Diabetes		20 (n = 8)	20 (n = 8)	60 (n = 24)	p = 0.001	-
		OHA	15 (n = 6)	15 (n = 6)	45 (n = 18)	NS	-
		Insulin	5 (n = 2)	5 (n = 2)	15 (n = 6)	NS	-
Total number of CVRFs			4.2 ± 0.8	4.3 ± 0.8	4.4 ± 0.9	NS	-
Infarct-related vessel (LAD/LCX/RCA)			19/8/13	20/10/11	20/8/12	NS	-
PTCA/Stent at the time of AMI			40/40	40/40	40/40	NS	-
Ejection fraction (%)			45 ± 10	43 ± 11	40 ± 10	NS	69 ± 9
Medication (%)							-
Aspirin			100	100	100	NS	-
Clopidogrel			100	100	100	NS	-
ACE inhibitor or AT II blocker			100	100	100	NS	-
Beta-blocker			100	100	100	NS	-
Aldosterone Antagonist			25	25	30	NS	-
Statin			100	100	100	NS	-

Ischemic heart disease (IHD), Oral hypoglycaemic agent (OHA), Coronary artery disease (CAD), Percutaneous transluminal coronary angioplasty (PTCA), Creatine kinase (CK), Left anterior descending coronary artery (LAD), Left circumflex artery (LCX), Right coronary artery (RCA), Coronary artery disease (CAD), Cardiovascular risk factors (CVRFs), Acute myocardial infarction (AMI), None significant (NS).

### Functional activity of BM-CPCs

**Figure 1:**
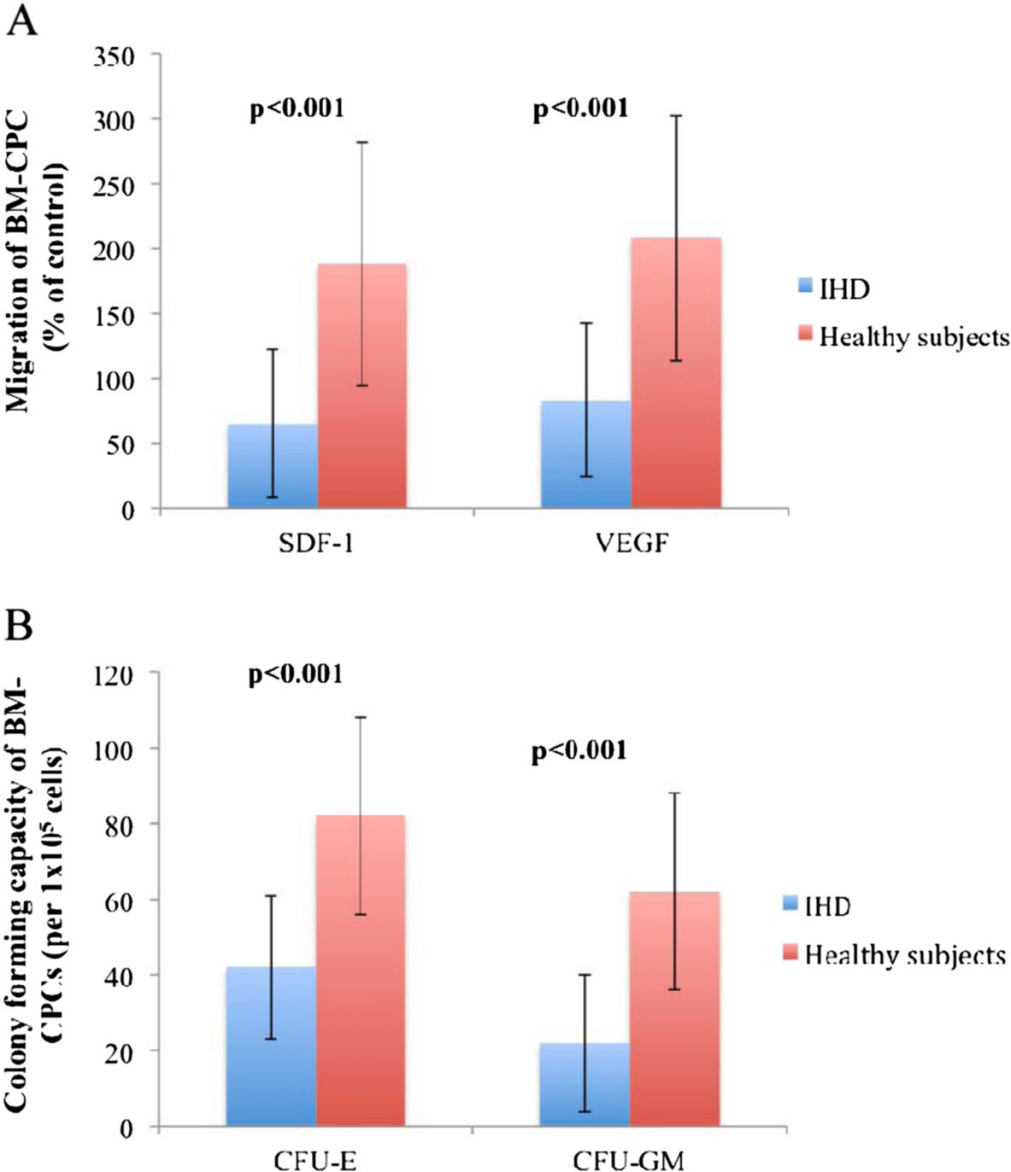
A and B: The colony-forming capacity and migratory response to stromal cell-derived factor 1 as well as vascular endothelial growth factor of BM-CPCs were reduced in patients with IHD compared to control group.

**Figure 2:**
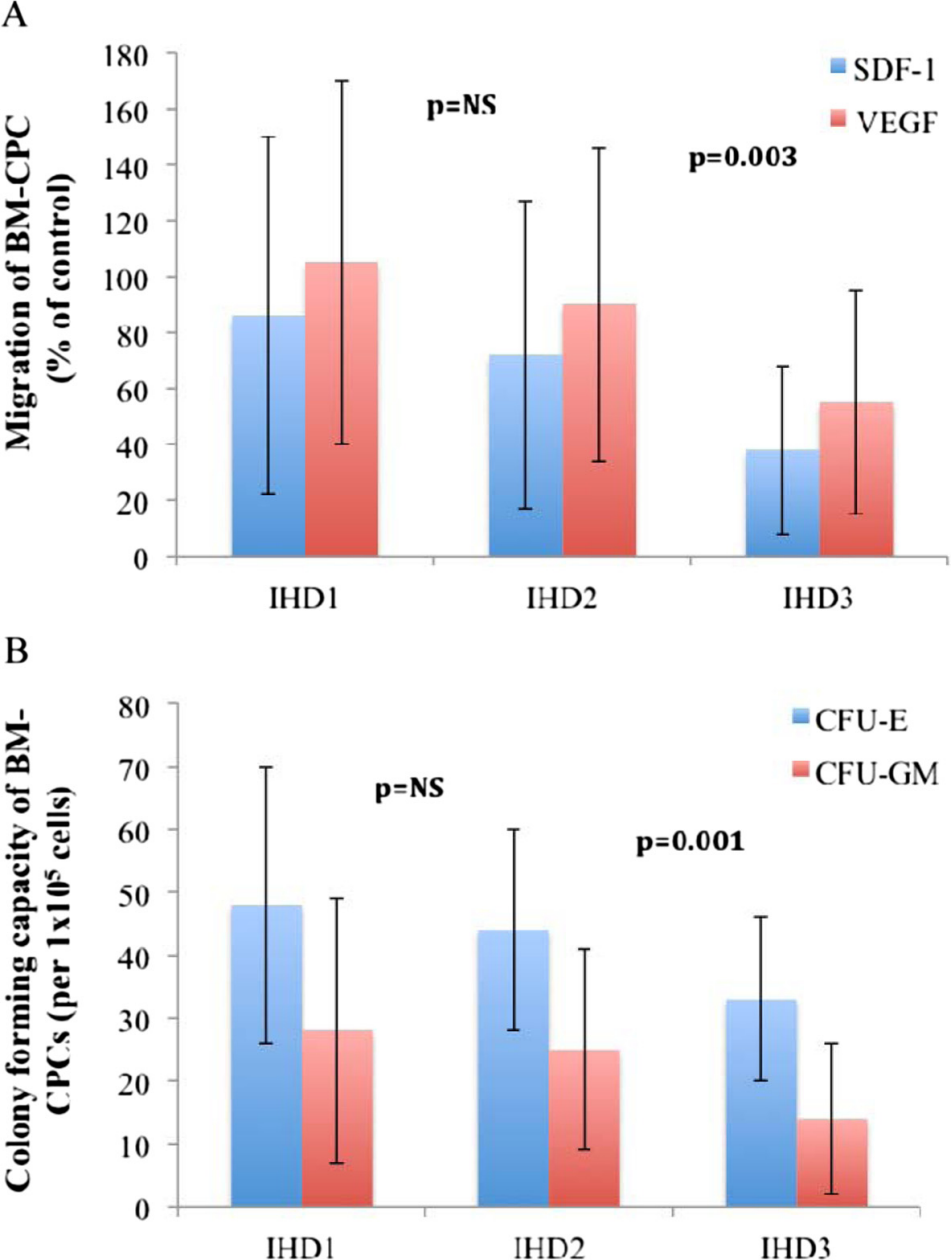
**A and B: The migratory -and colony forming capacities of BM-CPCs were significantly impaired in patients with IHD3 compared to IHD1 and IHD2.** In contrast, there were no significant changes in functional activity of BM-CPCs between the patients with IHD2 and IHD1.

The functional capacities of BM-CPCs were measured by migratory- and colony forming assay in 120 IHD patients as well as in 40 healthy subjects on day 1 of admission. The migratory response to stromal cell-derived factor 1 (SDF-1: p < 0.001) as well as vascular endothelial growth factor (VEGF: p < 0001) of BM-CPCs was impaired in patients with IHD compared to control group. Likewise, the colony-forming capacity (CFU-E: p < 0.001, CFU-GM: p < 0.001) was reduced in patients with IHD compared to control group. (Figure 1A and 1B) Furthermore, we found that the migratory -and colony forming capacities of BM-CPCs were significantly impaired in patients with IHD3 compared to IHD1 (VEGF: p < 0.001, SDF-1: p < 0.001; CFU-E: p < 0.001, CFU-GM: p < 0.001) and to IHD2 (VEGF: p = 0.003, SDF-1: p = 0.003; CFU-E: p = 0.001, CFU-GM: p = 0.001). In contrast, there were no significant differences in the functional activity of BM-CPCs between the patients with IHD2 and IHD1 (VEGF: p = 0.8, SDF-1: p = 0.9; CFU-E: p = 0.1, CFU-GM: p = 0.1). (Figure 2A and 2B)

### Impact of DM on the functional activity of BM-CPCs in patients with IHD

**Figure 3:**
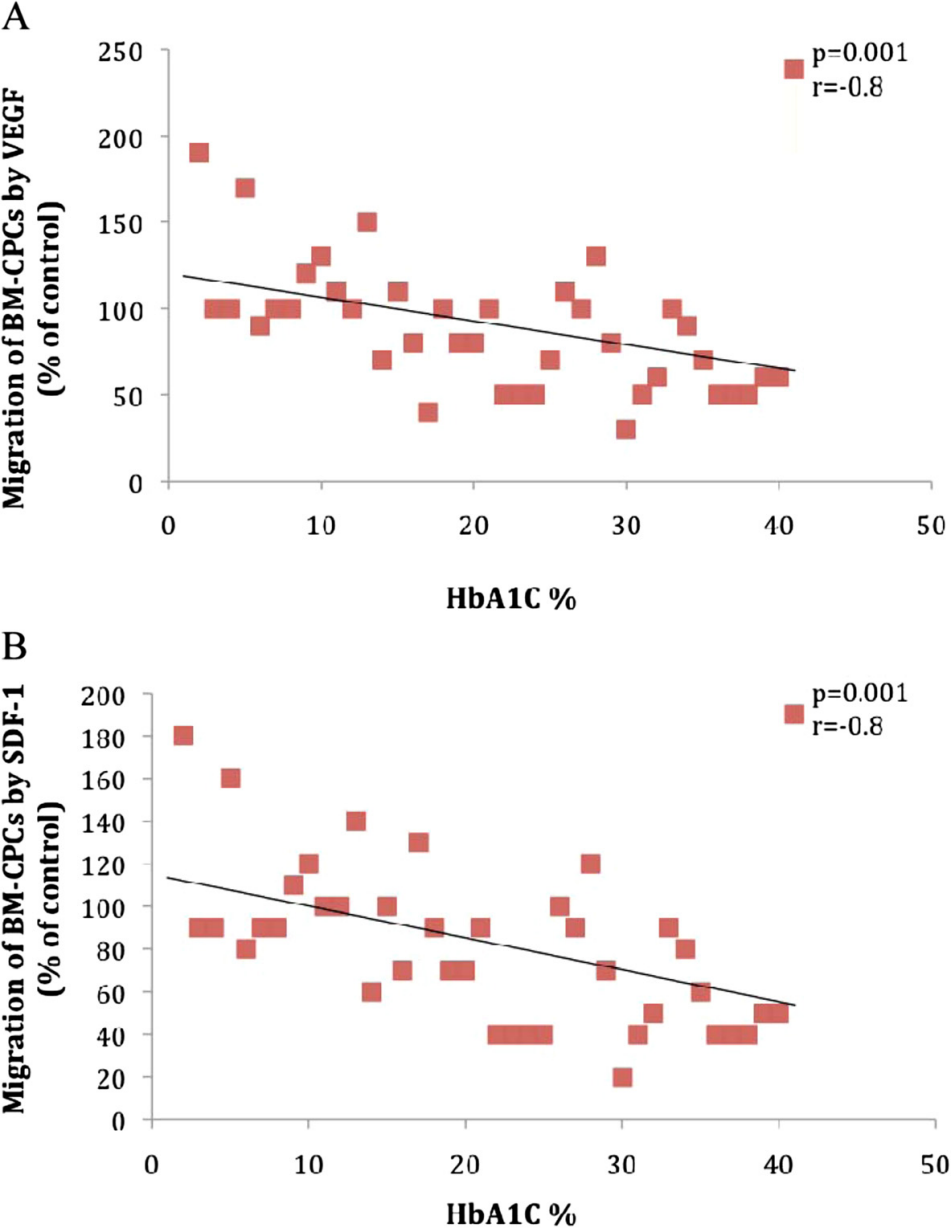
A and B: The migratory response to VEGF and SDF-1 correlates inverse to the level of HbA1c in patients with DM.

We investigated the correlation between DM (n = 40) and functional activity of BM-CPCs in patients with IHD. We observed a significant inverse correlation between levels of HbA1c and the migratory (VEGF: p < 0.001, r = −0.8 SDF-1: p < 0.001, r = −0.8) and colony forming capacities (CFU-E: p = 0.001, r = −0.7, CFU-GM: p = 0.001, r = −0.6) of BM-CPCs. (Figure 3. A, 3B, 4A and 4B) The functional activity of BM-CPCs was significantly reduced in DM patients with HbA1c >7% (n = 20) as compared to DM patients with HbA1c <7% (n = 20). (VEGF: p < 0.001, SDF-1: p < 0.001; CFU-E: p < 0.001, CFU-GM: p < 0.001) (Figure 5A and 5B)

**Figure 4:**
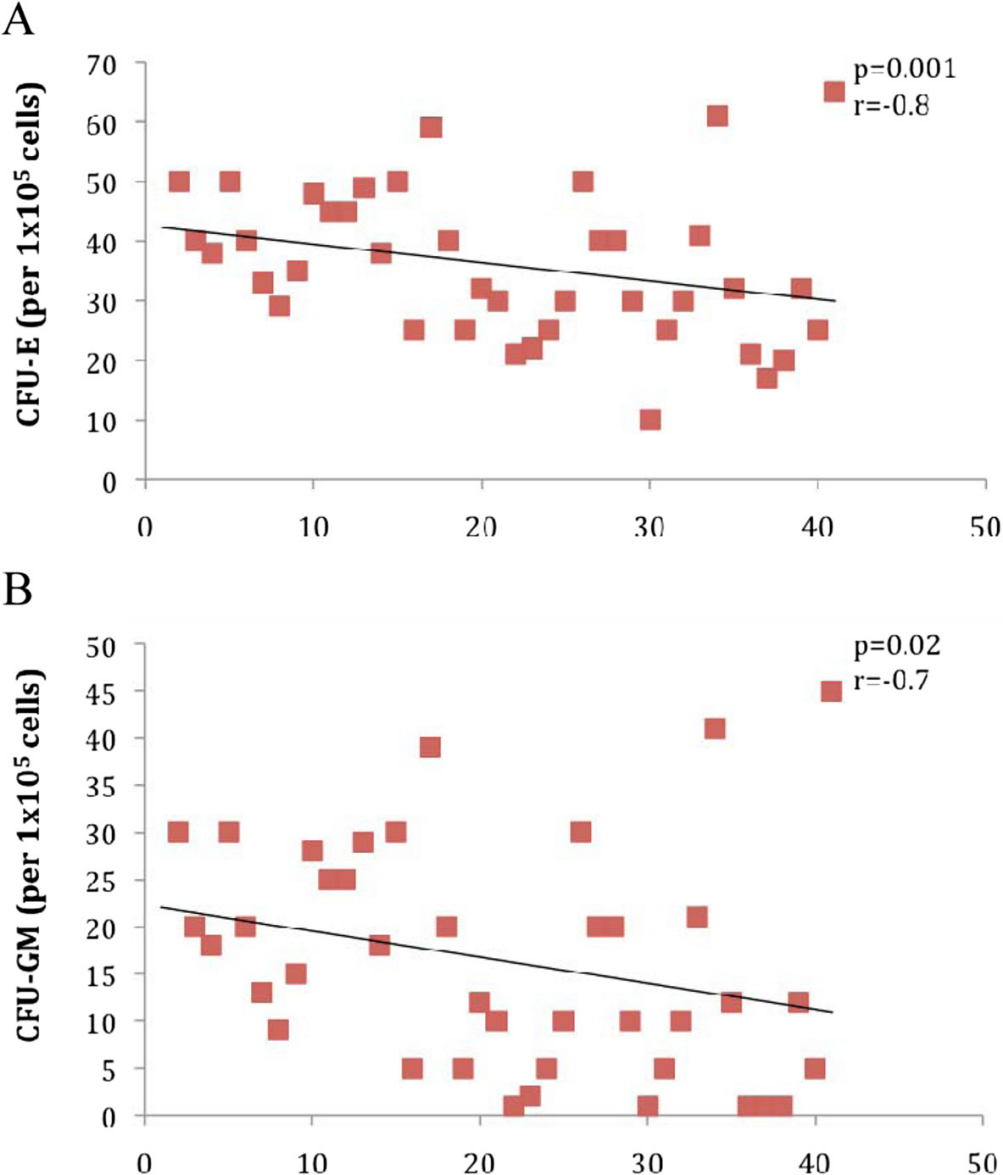
A and B: The colony forming capacitiy correlates inverse to the level of HbA1c in patients with DM.

**Figure 5:**
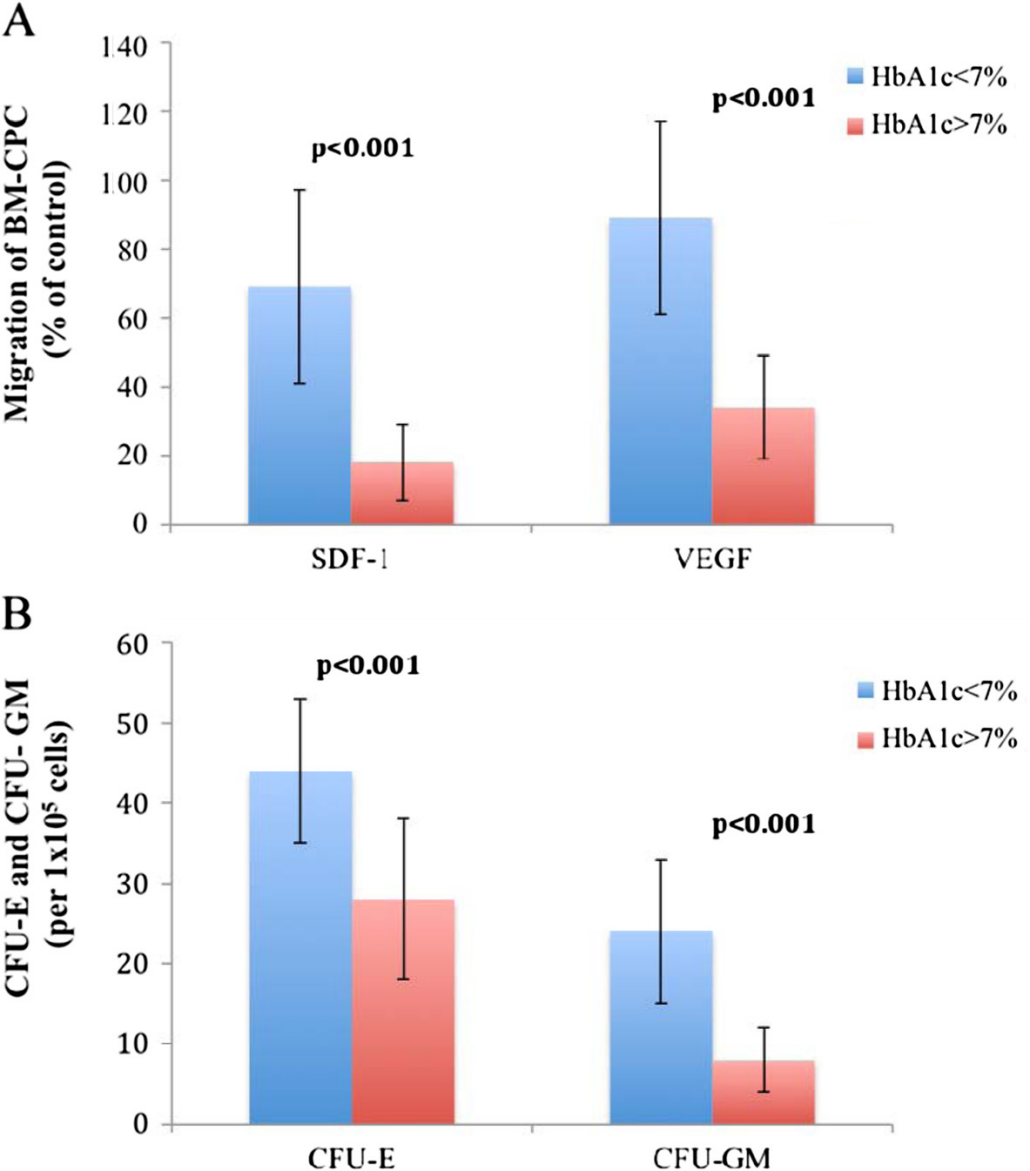
A and B: The migration and colony forming capacities of BM-CPCs are significantly reduced in patients with HbA1c >7% compared to patients with HbA1c <7%.

## Discussion

In this study we examined the influence of the number of diseased coronary arteries on the functional activity of BM-CPCs in patients with IHD.

Coronary artery disease results from a chronic inflammatory disease of the vascular wall and leads to vessel occlusion and organ damage. [17,18] Despite intense efforts to determine the pathogenesis of atherosclerosis, this process remains poorly understood. Reports suggest that risk factors and a genetic predisposition together induce inflammatory processes that lead to cell damage and impair regeneration within the vessel wall. [19,20] Since resident endothelial cells infrequently proliferate, [21] it has been postulated that there are other sources of vascular replenishment in response to continuous damage [22]. Circulating progenitor cells derived from bone marrow circulate in the peripheral blood and have been implicated in neoangiogenesis after tissue ischemia has occurred. [23-26] BM-CPCs is another population of progenitor cells that has also been shown to have therapeutic potential in the pool of progenitor cells circulating within the blood. BM-CPCs are capable of proliferating and differentiating into endothelial cells and are therefore ideal candidates for vascular regeneration. [27,28] Experimental and clinical studies suggest that the systemic application or mobilization of stem cells and progenitor cells beneficially influences the repair of endothelial cells after injury and the progression of atherosclerosis. [29-35] Previous studies demonstrate that risk factors for coronary artery disease correlate with number and functional activity of BM-CPCs. [8] Not only is the quantity of BM-CPCs altered, but their function is also modified by ischemic conditions and therapeutic interventions. [16] Migration is essential for the stem/progenitor cells to invade the ischemic tissue. SDF-1 and VEGF are both profoundly upregulated in hypoxic tissue [36,37] and thus represent physiologically relevant chemoattractants for the recruitment of circulating progenitor cells to sites of ischemia. Indeed, intramuscular injection of the chemoattractant chemokine SDF-1 has recently been shown to increase the number of incorporated BM-CPCs and to improve neovascularization in vivo. [38] Thus, the migratory response toward SDF-1 may indeed play a crucial role for integration of BM-CPCs in ischemic tissue. In addition, the accumulation of cardiovascular risk factors or an increased overall risk is associated with impaired colony forming activity of CPCs in healthy men. [9] Werner et al. [18] identified a significant association between increasing numbers and functional activities of BM-CPCs and decreased risk of major cardiovascular event and hospitalization in patients with coronary artery disease. In humans, a small-scale study suggests that there is a higher incidence of restenosis and revascularization in patients with reduced levels of BM-CPCs than in patients with increased numbers. [18,39] Recently we showed that Intracoronary transplantation of autologous BMCs may enhance and prolong the mobilisation as well as functional activity of BM-CPCs in PB in patients with ischemic heart disease and this might increase the regenerative potency in IHD. [40-42] Moreover, the mobilization of CD34^+^ and CD133^+^ BM-CPCs is further impaired by DM in patients with IHD. [43,44] However, it is unknown whether the functional activity of BM-CPCs relates with the number of diseased coronary arteries in patients with IHD. We demonstrated in our study, that the functional activity of BM-CPCs was significantly impaired in patients with IHD3 compared to IHD2 and IHD1. Diabetes mellitus is associated with both an increased risk of atherosclerotic disease and poor outcome after vascular occlusion. The clinical severity of vascular occlusive disease in diabetics has in part been attributed to impaired collateral vessel development [45]. Extensive studies have shown that the numbers of circulating angiogenic cells are significantly lower in type II diabetes, and their angiogenic potential is also dramatically diminished. These cells display defective adhesion to the endothelium, reduced proliferation rate, and impaired ability to create new vascular structures. [11,12,46] On the basis of these findings, it is tempting to speculate that the decrease of functional activity of BM-CPCs by DM leads to progression of atherosclerosis and increases the number of diseased coronary arteries in patients with IHD. In line with this hypothesis we observed in our study a significant higher incidence of DM in patients with IHD3 compared to IHD2 and IHD1. Furthermore, we demonstrated that the functional activity of BM-CPCs inversely correlated with the level of HbA1c in IHD patients with DM. The functional activity of BM-CPCs in DM patients with HbA1c > 7% was significantly reduced compared to DM patients with HbA1c <7%. Recent studies have shown that the PPARϒ agonist pioglitazone treatment increases the number and function of BM-CPCs in type 2 DM patients with coronary artery disease. [47,48] Improved levels of HbA1c by pharmacological therapy may lead to increase of BM-CPCs mobilization and functional activity and thereby may enhance the vascular regeneration in IHD patients with DM.

## Conclusion

In the present study we could demonstrate that the functional activity of BM-CPCs was impaired in patients with IHD. This impairment correlates with increase of the number of diseased coronary arteries. Moreover, the regenerative capacity in ischemic tissue of BM-CPCs further declines in IHD patients with DM.

## Competing interests

The authors have no competing interests to declare.

## Authors’ contributions

All authors have read and approved the final manuscript. The specific contributions of each author are; IBT, RGT and CAN conceived, designed and directed the entire study, interpreted all data and wrote the manuscript. LP, NA, CHT, IA, SK, JO, HS, SL, TH, SD, RA, HI and GA collected the preliminary data, participated in the study design, interpretation of the data, revision of paper.
